# Intradural Lumbar Disc Herniation with Focal Intraspinal Gas and Pseudocystic Morphology: Radiologic-Operative-Pathologic Correlation

**DOI:** 10.1055/a-2984-6389

**Published:** 2026-11-03

**Authors:** Juan Carlos Gonzalez-Gutierrez, Ali Ahmad, Ziad Ismael, Jochen Tuettenberg

**Affiliations:** 1 Department of Neurosurgery 68183 Klinikum Idar-Oberstein GmbHIdar-ObersteinRhineland-PalatinateGermany

**Keywords:** intradural disc herniation, lumbar disc herniation, spinal canal gas, vacuum phenomenon, cauda equina, case report

## Abstract

Intradural lumbar disc herniation is rare and frequently recognized only during surgery because its clinical and imaging features may resemble other intradural lesions. A 71-year-old man presented with progressive left-sided lumbosciatica without neurological deficit. Magnetic resonance imaging showed a pseudocystic intradural lesion at L4–L5 with marked displacement of the cauda equina, and computed tomography demonstrated focal gas in the ventral spinal canal at the same level. Extradural exploration did not reveal disc material sufficient to explain the compression. Dorsal durotomy released air and exposed a pseudocystic lesion containing disc material. After resection, a ventral dural defect communicating with the disc space was visualized and reinforced with a fibrin sealant patch. Histopathology confirmed degenerated disc tissue with granulation tissue and pseudocystic fibrous tissue. The patient remained neurologically intact and experienced complete resolution of the presenting radicular symptoms. Gas-containing cystic intradural disc herniation has been reported previously; the value of this case is the concordance of routine magnetic resonance and computed tomography findings with air release, direct visualization of the migration route, and histopathologic confirmation. Focal intraspinal gas does not establish intradural migration by itself, but in the appropriate anatomic setting it can strengthen diagnostic suspicion and surgical planning.

## Introduction

Intradural lumbar disc herniation (ILDH) is an uncommon form of disc disease in which disc material penetrates the dura and enters the intradural compartment. It accounts for approximately 0.04 to 0.33% of lumbar disc herniations and occurs most often at L4–L5. 1 2

Since Dandy’s first description in 1942, 3 preoperative recognition has remained difficult because clinical findings resemble conventional lumbar disc disease, whereas imaging may suggest an intradural tumor or cyst. 4 5 6 7 Dense attachment of the posterior longitudinal ligament (PLL) to the ventral dura is considered central to transdural migration. 8 9 Gas-associated and cystic ILDHs have also been described. 10 11 12 We report an L4–L5 lesion that integrates noninvasive imaging, operative visualization of a communicating ventral defect, and histopathologic confirmation.

Recognition before or during the initial exposure has practical consequences. A surgeon expecting only an extradural discectomy may not be prepared for durotomy, intradural rootlet dissection, or management of a ventral dural defect. Conversely, interpreting the lesion only as a neoplasm may obscure its relationship to the disc space.

## Case Report

A 71-year-old man presented with a 4-week history of progressively worsening low back pain radiating along the lateral and anterior left thigh into the anterior lower leg and ankle. During the days before admission, pain markedly restricted mobility. He denied numbness, paresthesias, saddle anesthesia, bowel or bladder dysfunction, and subjective weakness.

Neurologic examination showed full strength in both lower extremities, symmetric reflexes, absent Babinski responses, and no objective sensory deficit. The Lasègue test was negative bilaterally. Gait was mildly antalgic because of pain, but heel and toe walking were preserved.

Lumbar magnetic resonance imaging (MRI) demonstrated a pseudocystic intradural lesion immediately dorsal to the L4–L5 disc, with marked displacement and compression of the cauda equina. Computed tomography (CT) showed focal gas in the ventral spinal canal at the same level. The close relationship among the lesion, disc space, and gas raised preoperative suspicion for ILDH, although other intradural lesions remained in the differential diagnosis ( Fig. 1A–D ).

**Fig. 1:**
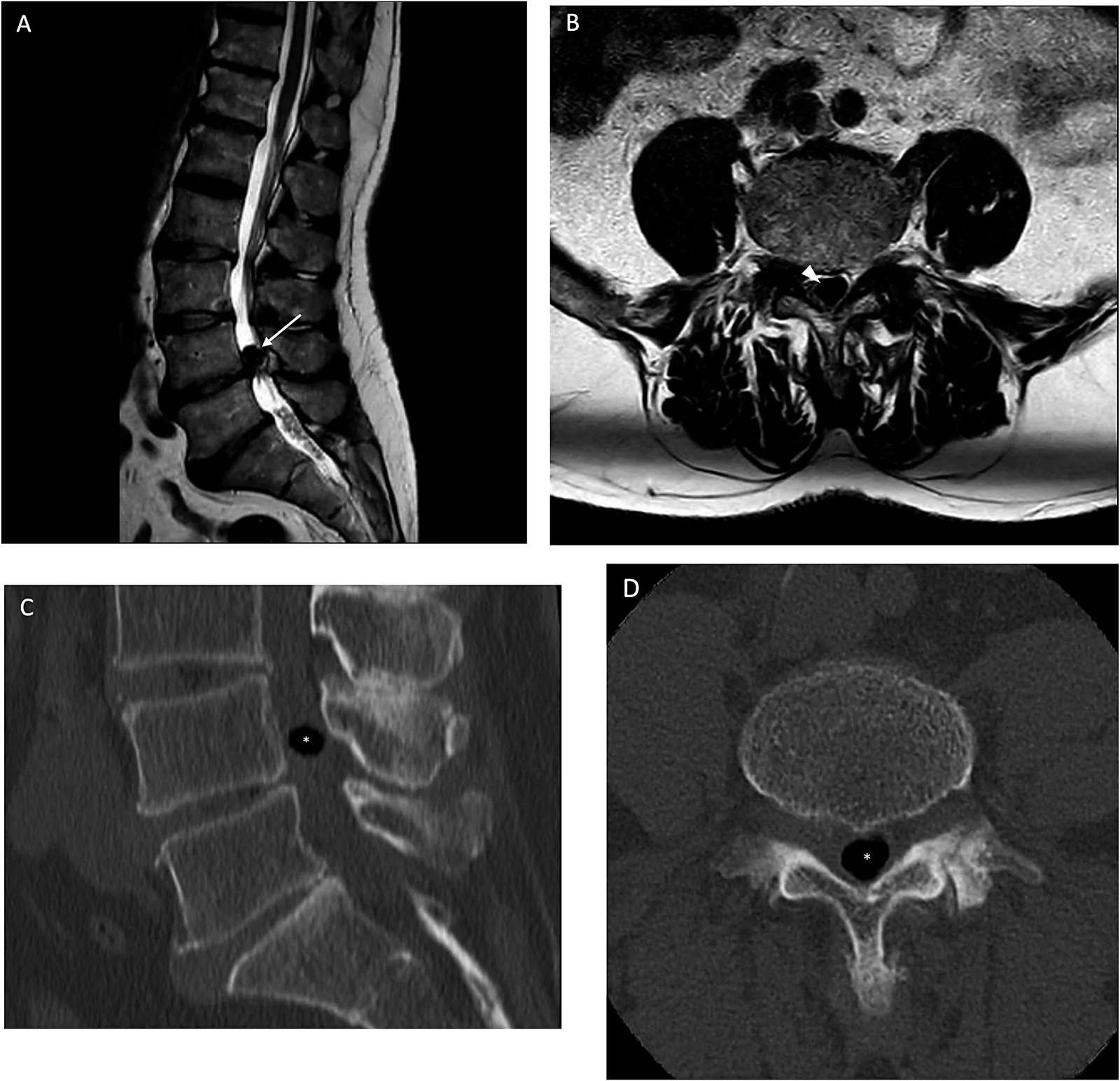
Preoperative imaging. ( **A** ) Sagittal T2-weighted magnetic resonance image showing a pseudocystic intradural lesion immediately dorsal to the L4–L5 disc with cauda equina compression (arrow). ( **B** ) Axial T2-weighted image showing displacement of the cauda equina rootlets by the lesion (arrowhead). ( **C** ) Sagittal computed tomography image showing focal gas in the ventral spinal canal at L4–L5 (asterisk). ( **D** ) Axial computed tomography image showing focal intraspinal gas at the same level (asterisk).

Because symptoms were severe and progressive, microsurgical decompression was performed through a left L4–L5 hemilaminectomy and flavectomy. The ventral dura was densely adherent to the underlying disc space and was difficult to mobilize. Extradural exploration did not reveal a conventional extrusion sufficient to explain the compression on MRI. The disc space was incised and several fragments were removed, yet the intradural mass remained palpable. This imaging–operative discrepancy prompted dorsal durotomy.

Air escaped immediately after dural opening. Intradural exploration revealed a pseudocystic lesion densely adherent to adjacent rootlets. The lesion was separated under the microscope and opened, exposing disc material that was removed piecemeal. Complete excision exposed a ventral dural defect directly communicating with the disc space. Because primary ventral suturing was not technically feasible, the defect was reinforced with a fibrin sealant patch. The dorsal durotomy was closed watertight with 6–0 nonabsorbable monofilament suture ( Fig. 2A–D ). Histopathology demonstrated intervertebral disc tissue with granulation tissue and fibrous connective tissue containing pseudocystic spaces, without malignancy.

**Fig. 2:**
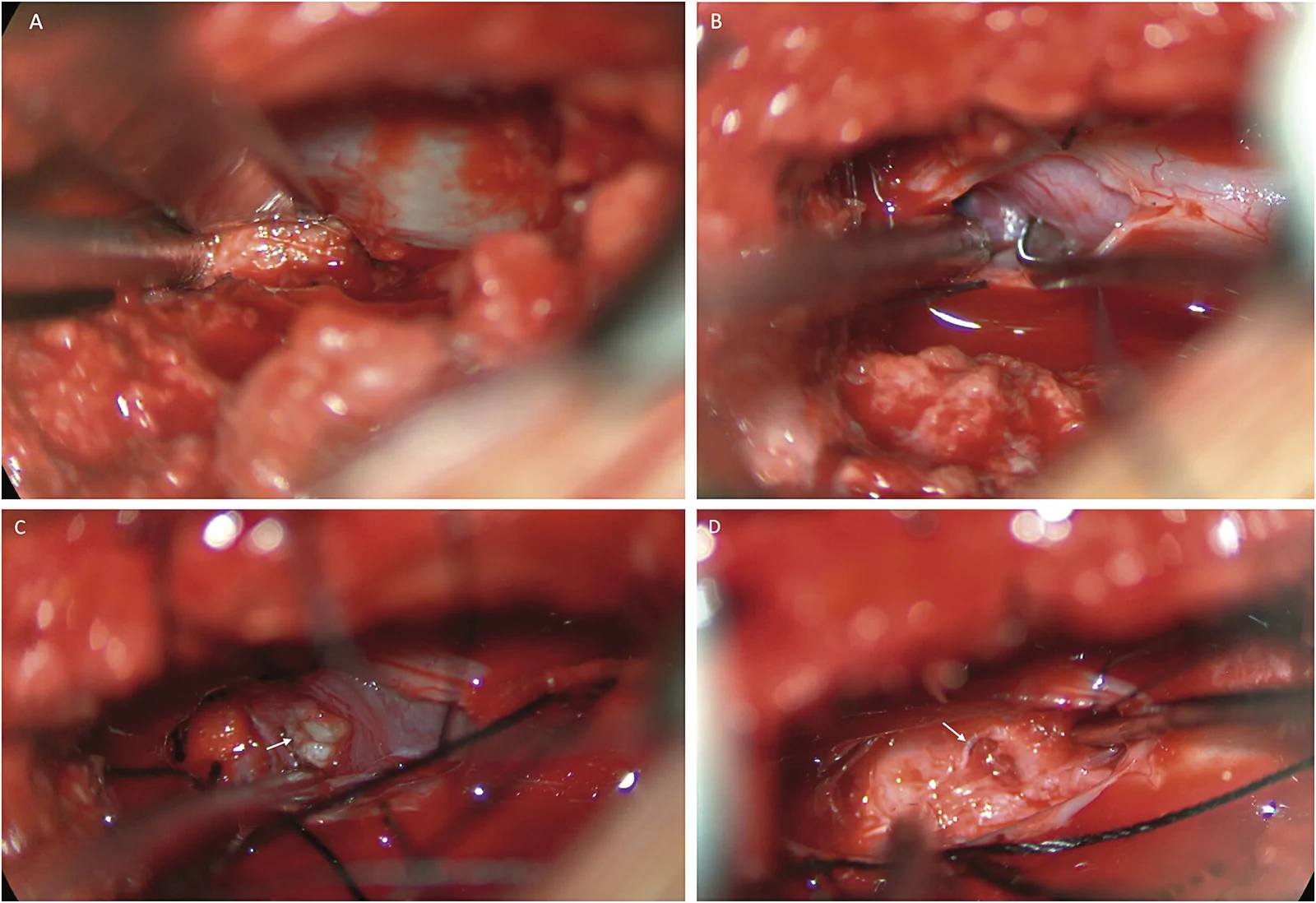
Intraoperative findings. ( **A** ) Dense adhesion between the ventral dura and underlying disc space, limiting mobilization of the dural sac. ( **B** ) Intradural compartment after dorsal durotomy. ( **C** ) Pseudocystic intradural lesion containing disc material (arrow) and displacing adjacent rootlets. ( **D** ) Ventral dural defect communicating with the disc space after lesion removal (arrow).

The patient developed no new neurologic deficit. Radicular pain improved markedly on postoperative day 1. A transient exacerbation on day 2 responded to a short course of dexamethasone. At discharge, pain had decreased from 9/10 to 3/10 and wound healing was uneventful. At the 3.5-month follow-up, the preoperative left-sided radicular symptoms had resolved completely. Temporary right S1-distribution pain during recovery resolved spontaneously. Follow-up MRI showed satisfactory decompression and no residual intradural lesion ( Fig. 3A,B ).

**Fig. 3:**
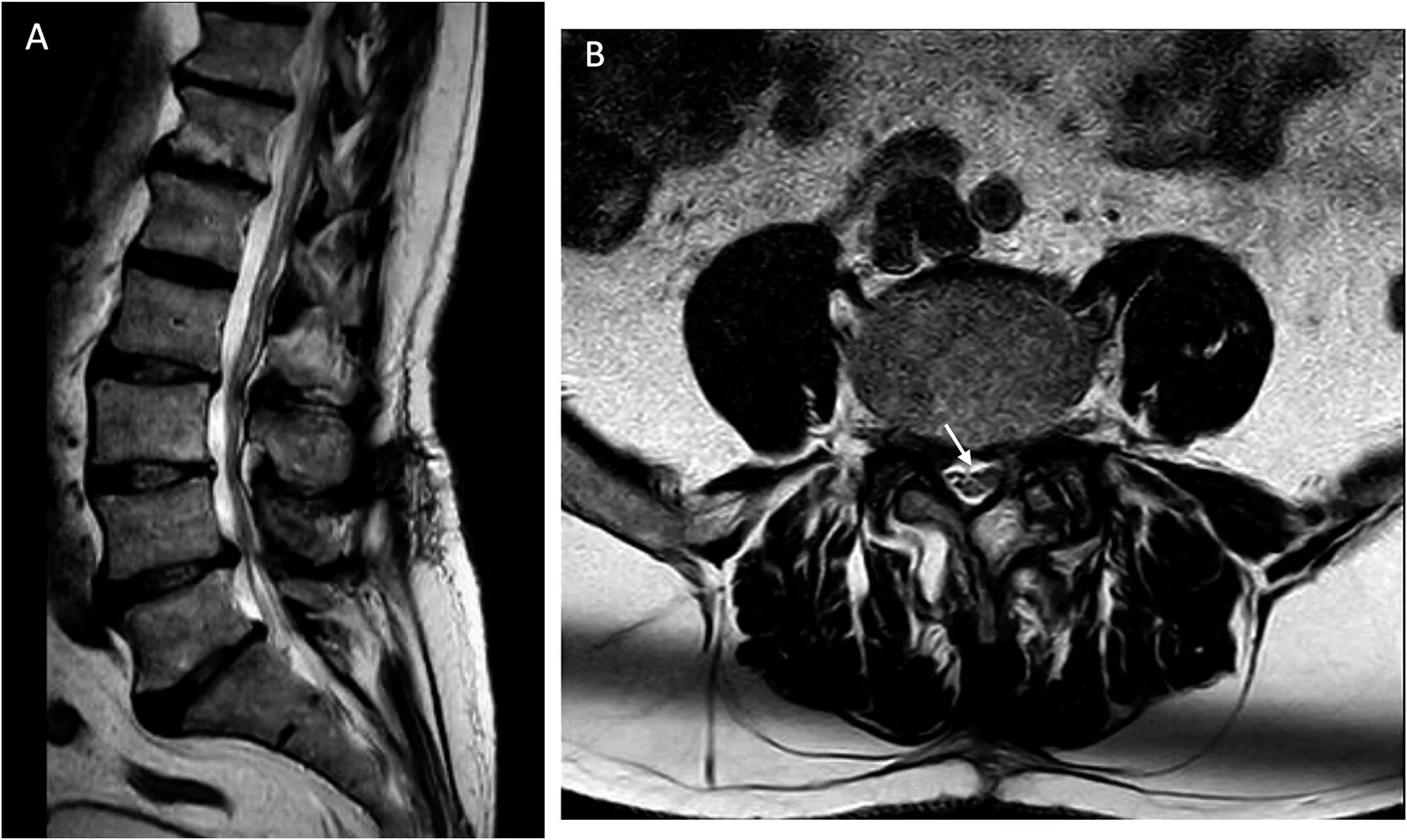
Postoperative imaging. ( **A** ) Sagittal T2-weighted magnetic resonance image showing adequate decompression at L4–L5 without a residual intradural lesion. ( **B** ) Axial T2-weighted image showing restoration of the intradural space and satisfactory neural decompression (arrow).

## Discussion

The prevailing mechanism of ILDH is chronic adhesion among the annulus, PLL, and ventral dura, followed by penetration of the adherent tissue complex by disc material. 8 9 Congenital attachments, inflammation, degeneration, previous surgery, trauma, and repetitive mechanical irritation have been proposed as contributing factors. Yildizhan et al found the strongest ventral dura–PLL attachment at L4–L5 in an anatomic study. 8 Dense ventral adhesions and direct visualization of a dural defect communicating with the disc space support this mechanism in our patient.

Preoperative MRI clues include abrupt PLL interruption, extension beyond the PLL, a hawk-beak configuration, peripheral rim enhancement, and displacement of cauda equina rootlets. 4 5 6 7 None is consistently present. In this case, MRI established the intradural location and neural compression, whereas CT identified focal gas contiguous with the affected disc level. CT could not, by itself, establish that the gas was intradural. The combined findings were nevertheless more informative than either modality alone. A structured review of the lesion’s relationship to the PLL, dura, rootlets, disc space, and any adjacent gas is therefore preferable to reliance on a single named sign. Signal intensity also varies with fragment hydration and degeneration. When contrast-enhanced imaging is obtained, peripheral enhancement can represent reactive granulation around an avascular fragment; however, this pattern is not specific and may overlap with tumor, inflammation, or a sequestrated extradural fragment.

The diagnostic relevance of gas is not unique to this case. Hidalgo-Ovejero et al described ILDH associated with epidural gas, 10 11 and Sugiura et al reported a gas-containing cystic L1–L2 ILDH whose communication with the disc was confirmed by discography. 12 Gas is thought to originate from a degenerative vacuum phenomenon and migrate through annular, PLL, and dural defects. The present report adds concordant evidence at L4–L5: a pseudocystic intradural mass on MRI, focal intraspinal gas on CT, immediate air release at durotomy, a directly visualized migration route, and pseudocystic tissue surrounding disc material on histology. This convergence, rather than gas alone, is the principal teaching point. Gas in the spinal canal is most often a manifestation of degeneration and may be epidural or associated with a facet cyst; much less commonly, gas can accompany infection or an invasive procedure. Its interpretation therefore requires clinical context and precise anatomic correlation.

Unlike typical extradural lumbar disc herniation, for which conservative management is often appropriate in the absence of progressive weakness or cauda equina syndrome, the natural history of ILDH is less predictable. ILDH may present with severe radicular pain, cauda equina symptoms, or a tumor-like intradural mass, and rootlets can be displaced or adherent to the fragment. Spontaneous resorption has been reported, including the recurrent ILDH described by Sakai et al. 13 However, this evidence remains limited to rare case reports and does not define reliable criteria for observation. In our patient, surgery after 4 weeks of symptoms was chosen because pain was severe and progressive, mobility was markedly limited, imaging showed substantial cauda equina compression, and diagnosis of the intradural lesion was required.

Mut et al classified intradural disc herniations as type A when the fragment lies within the dural sac and type B when it extends into a nerve-root dural sheath. 14 The present lesion was type A. This distinction has practical implications. Type A lesions usually require dorsal durotomy, intradural exploration, microsurgical dissection from cauda equina rootlets, and inspection of the ventral dural defect. Type B intraradicular lesions may carry greater risk of direct nerve-root injury because disc material extends into the dural sleeve of an individual nerve root and may require opening of the involved root sheath with more selective dissection. Thus, the classification may not change the initial posterior exposure, but it can influence the extent of intradural dissection, risk counseling, and neural preservation strategy. The pseudocystic morphology in our case also broadened the differential diagnosis, and histologic confirmation remained important. 15

The key intraoperative warning sign was the mismatch between severe compression on imaging and the absence of a corresponding extradural fragment. Similar reports emphasize inspection and palpation of the thecal sac and intradural exploration when this discrepancy persists. 16 17 Continued blind extradural manipulation may be ineffective and risks neural injury. During durotomy, adherent rootlets require meticulous dissection. Intraoperative ultrasound, when available, may help confirm that a persistent mass is intradural and guide the safest dural opening. The ventral defect should be sought because it confirms the migration route and informs closure. When direct suturing is unsafe, patch reinforcement combined with watertight dorsal closure is a pragmatic option, with close postoperative surveillance for cerebrospinal fluid leakage.

This report is limited by its single-case design and cannot establish the diagnostic accuracy of gas or any individual MRI sign. The exact intradural location of the gas was confirmed only at surgery, and this observation does not establish a general causal relationship between vacuum gas and transdural migration. The report also cannot compare patch reinforcement with primary ventral suturing or other closure strategies. Nevertheless, the complete radiologic–operative–pathologic sequence offers a reproducible diagnostic and surgical reasoning framework for an uncommon lesion.

## Conclusion

ILDH should be considered when an intradural lumbar lesion is contiguous with a diseased disc and focal intraspinal gas, or when extradural findings do not explain the compression seen on imaging. Concordant MRI, CT, and operative and histopathologic findings—not gas alone—can strengthen diagnosis and guide safe intradural exploration.

## Declaration of GenAI Use

OpenAI ChatGPT, a generative artificial intelligence tool, was used to assist with English-language editing, manuscript structure, and reference checking. The tool was not used to generate clinical data, images, diagnoses, or scientific conclusions. All authors critically reviewed, edited, and approved the final manuscript and take full responsibility for its content.
